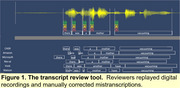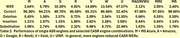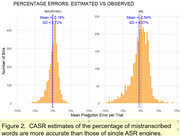# Advances in automated speech transcription during cognitive testing

**DOI:** 10.1002/alz70861_108225

**Published:** 2025-12-23

**Authors:** David L. Woods, Michael Blank, Kristin Geraci, Timothy J Herron, Isabella Jaramillo, Andrew L Boghossian, David K Johnson, Evan L Richards, Peter Pebler, Kathleen Hall

**Affiliations:** ^1^ Neurobehavioral Systems, Inc, Berkeley, CA USA; ^2^ Veterans Affairs Northern California Health Care System, Martinez, CA USA; ^3^ UC Davis Alzheimer's Disease Center, Walnut Creek, CA USA

## Abstract

**Background:**

Accurate automatic speech recognition (ASR) is essential for the objective analysis of speech during cognitive testing. Here, we describe the performance of seven individual ASR engines and selected consensus ASR (CASR) engine combinations when analyzing tokens generated by 453 randomly selected older subjects **(mean age 60.5 years, 52% non‐white)** who completed normative enrollment testing for the California Cognitive Assessment Battery (CCAB).

**Method:**

High‐quality (24‐bit 48 kHz) digital recordings were captured via noise‐canceling head‐mounted microphones during at‐home testing. Recordings were downsampled and transcribed using **seven ASR engines**: Amazon Transcribe, Google Cloud, Microsoft Azure, IBM Watson, Rev.ai, Vosk, and UWP (Windows real‐time transcription). This analysis focuses on **three discursive speech tasks**: two logical memory stories and a picture description with delayed recall. Custom grammars consisting of the words in the logical memory stories and key picture elements were used with each engine. Transcripts containing **412,741 words** were reviewed and corrected using the CCAB's transcript review tool (Figure 1). The evidence for each utterance was estimated from **lookup tables** relating transformed engine confidence to accuracy for each ASR engine. All speech—including extraneous utterances (e.g., “I forgot that!!”) and subvocalizations—was retained. Word‐level evidence was estimated from lookup tables mapping transformed engine confidence values to empirical accuracy for each ASR engine. For CASR, word alignments were computed using the Levenshtein algorithm, and the total evidence (including for null entries and dysfluencies) was summed across engines to identify the most probable consensus word.

**Result:**

Word error rates (WERs) for single ASR engines ranged from 3.68% to 47.67%. CASR analysis incorporating all engines **reduced the WER to 2.12%** (Table 1). In addition, CASR‐derived confidence scores correlated more strongly with transcription accuracy (r = 0.75) than those of the best individual engine (r = 0.47) and more accurately predicted transcription errors (Figure 2). CASR also mitigated transcription biases associated with race and gender.

**Conclusion:**

CASR achieves near‐human transcription accuracy, flags potentially unreliable transcripts, and reduces demographic bias, demonstrating its utility and robustness in high‐stakes cognitive assessments.